# Inequalities in Wellbeing in Lebanese Children and Different Refugee Subpopulations: A Multidimensional Child Deprivation Analysis

**DOI:** 10.1007/s12187-023-10040-2

**Published:** 2023-06-06

**Authors:** Zeina Jamaluddine, Gloria Safadi, Alexandra Irani, Nisreen Salti, Jad Chaaban, Sawsan Abdulrahim, Alban Thomas, Hala Ghattas

**Affiliations:** 1 Center for Research On Population and Health, Faculty of Health Sciences, American University of Beirut, Beirut, Lebanon; 2 London School of Hygiene and Tropical Medicine, London, UK; 3 Applied Economics and Development Research Group, American University of Beirut, Beirut, Lebanon; 4 Department of Economics, American University of Beirut, Beirut, Lebanon; 5 Department of Health Promotion and Community Health, Faculty of Health Sciences, American University of Beirut, Beirut, Lebanon; 6 Paris-Saclay Applied Economics, University of Paris-Saclay, INRAE, AgroParisTech, Palaiseau, France; 7 Observatory of Rural Development, INRAE, Toulouse, France; 8 Arnold School of Public Health, University of South Carolina, Columbia, SC USA

**Keywords:** Child poverty, Child well-being, Multidimensional deprivation, Refugees

## Abstract

**Background and Objectives:**

This study constitutes the first attempt to describe the overlapping deprivations faced by Lebanese children (Lebanese) and that of the three sub-populations of refugees living in Lebanon: Palestinian refugees living in Lebanon, Palestinian refugees from Syria and Syrian refugees.

**Methods:**

Using data from the United Nations International Children's Emergency Fund (UNICEF) Household Survey 2016 (*n* = 10,555 Lebanese; 7,106 Palestinian refugees living in Lebanon; 2,768 Palestinian refugees from Syria and 5,891 Syrian refugee children aged 2 to 17 years old), we report on single and overlapping deprivations (at least two concurrent deprivations) using indicators related to survival (nutrition, health, water, sanitation and overcrowding), development (education) and protection (labor, exposure to violence and early marriage). Maternal education and geographical correlates of deprivation were explored using multivariable logistic regression models clustering for children in the same households.

**Main Results:**

In terms of co-occurrence of deprivations, Syrian refugees had the highest prevalence in all age groups (68.5%, 2-4y and 65.7%, 6-17y), followed by Palestinian refugees from Syria (46.2%, 2-4y and 45.5%, 6-17y), Palestinian refugees living in Lebanon (28.9%, 2-4y and 23.7%, 6-17y), with Lebanese children having the lowest prevalence (13.2%, 2-4y and 15.3, 6-17y). About half of Palestinian refugees from Syria and Syrian refugees (6-17y) were deprived in protection and housing. Education deprivation is of primary concern for Syrian children. Higher maternal education was consistently associated with lower odds of co-occurrence of deprivations among children aged 6-17y.

**Conclusion:**

This study highlights the importance of including refugee populations in reporting frameworks. This analysis additionally generates geographical and socio-economic profiles of the deprived children and identifies key deprivation areas of the affected sub-groups to inform effective policy design especially in light of the prevailing economic crisis.

**Supplementary Information:**

The online version contains supplementary material available at 10.1007/s12187-023-10040-2.

## Background

It is well established that the social, economic, and physical environments within which children grow are strong determinants of child health and wellbeing (Blair et al., 2010; Viner et al., 2012). Poverty and inequalities in access to education and health care, poor environmental conditions, and disparities by gender and race/ethnicity, amongst other factors, play essential roles in setting the socio-economic positions of children, potentially compromising their future wellbeing outcomes (Moore et al., 2015; World Health Organization, 2010).

Multidimensional approaches to poverty measurement are increasingly adopted as opposed to unidimensional approaches based on income or household expenditures (Ferrone & de Milliano, 2018). While monetary poverty remains important in measuring poverty, it does not fully capture children's experience of poverty in a household, as children could be living in a non-poor household and yet be deprived due to unequal intra-household allocation of resources between household members (Adetola & Olufemi, 2012). In addition, monetary poverty does not entirely reflect children’s experience of poverty, because a significant number of goods and services, which are essential for a child's development, are dependent on the availability of public resources and infrastructure (education, health, water and electricity networks), or can't be bought (household protection, involvement and engagement in society) (Ferrone & de Milliano, 2018). Hence, as income fails to sufficiently capture child poverty and deprivation, more comprehensive metrics that recognize the influence of multidimensional deprivation on child wellbeing are warranted (Abdu & Delamonica, 2018).

Sustainable development goal (SDG) Target 1.2 specifically mentions poverty "in all its dimensions", with the requirement that countries measure and report on multidimensional poverty. Child-specific wellbeing indices that focus on the child as the unit of analysis and consider key age-specific dimensions of deprivation can better inform effective program/policy design to address child wellbeing. The Multiple Overlapping Deprivation Analysis (MODA) tool, developed by United Nations International Children's Emergency Fund (UNICEF), adopts a comprehensive approach to child wellbeing and recognizes the multidimensional nature of child deprivation (De Neuborg et al., 2012; Ferrone & de Milliano, 2018). MODA builds on research in the field of multidimensional poverty such as the UNICEF Global Study on Child Poverty and Disparities and the OPHI Multidimensional Poverty Index (De Neuborg et al., 2012). Yet, it distinguishes itself from other wellbeing indices and tools by focusing on the child as the unit of analysis rather than the household (De Neuborg et al., 2012). MODA additionally adopts a life-cycle approach that incorporates the different needs of children at various stages of development, from early childhood to primary childhood and adolescence (Chzhen et al., 2014). Through its overlapping deprivation analysis approach, MODA generates geographical and socio-economic profiles of deprived children and identifies key deprivation areas of the affected sub-groups to inform effective policy design (Chzhen et al., 2016).

Studies have shown that children who experience multidimensional deprivation are more likely to experience poor health outcomes, including malnutrition, infectious diseases, and limited access to medical care. In addition, children living in deprived conditions are less likely to access educational opportunities, which may limit their potential and perpetuate intergenerational poverty. In several contexts, policymakers have leveraged the MODA framework to identify areas and households with the highest levels of deprivation, informing the development of targeted interventions aimed at reducing poverty and enhancing the wellbeing of children (Chzhen et al., 2018; De Milliano & Plavgo, 2018; Mahrt et al., 2020).

Maternal education has been put forward as one of the strongest determinants of child health and wellbeing (Güneş, 2015). Educated mothers have been shown to potentially have more comprehensive health knowledge, greater receptivity to health messages, and higher rates of health care utilization, ultimately improving their children’s wellbeing (Vikram et al., 2012). Likewise, structural factors such as a child's geographical area of residency could potentially be viewed as crucial in shaping a child's wellbeing outcomes. Studies corroborate that resource distribution, societal structural aspects and isolation are important for children’s experiences of deprivation and consequent wellbeing (Campos et al., 2018). The cumulative effect of these determinants is particularly concerning in early childhood, a critical period of growth and development (Kuruvilla, 2016).

### The Lebanese Context

Almost twelve years since the beginning of the Syrian conflict, Lebanon remains at the forefront of one of the worst humanitarian crises globally and hosts the largest number of refugees per capita in the world, representing almost one in five of its total population (UNDP, 2016). While an estimated 270,000 Palestinians refugees were already living in Lebanon, descendants of the refugee waves starting in 1948 (UNRWA, 2017), the Syrian crisis brought an additional 30,000 Palestinian refugees from Syria (UNRWA, 2019) and over 1.5 million Syrian refugees; almost half of whom are women and children (UNHCR et al., 2019). Despite the protracted nature of their presence in the country, refugee children have collectively faced structural and legal obstacles by the mere virtue of being non-nationals. As such, exclusionary policies have kept poverty levels high among refugees who have consequently relied on long-term humanitarian relief systems(Chaaban et al., 2010).

Since October 2019, Lebanon has been witnessing a severe economic crisis that led to a rapid currency depreciation and ensuing hyperinflation of 171.2% in 2022 (Central Administration of Statistics, 2023). This has been further exacerbated by both the COVID-19 pandemic and a historic explosion in its capital city Beirut. Estimates prior to these compounded crises indicate that nearly 65% of Palestinian refugees living in Lebanon (Chaaban et al., 2015) and 90% Palestinian refugees from Syria (Chaaban et al., 2015; UNHCR et al., 2019) were living on less than 6.8 USD per day and that 73% of Syrian refugees were living on less than 3.84 USD per day (Chaaban et al., 2015; UNHCR et al., 2019). In light of the current situation, poverty levels are estimated to further rise for refugees as well as for vulnerable Lebanese sub-populations, reaching levels as high as 50% poverty for Lebanese nationals, with knock-on effects on children’s wellbeing and potential for development (WFP, 2020).

Due to their legal status, refugees are classified as non-national residents in the countries where they seek refuge and are therefore mostly excluded from regular data collection processes that national governments undergo to produce statistics on their citizens. As a result, refugees are missed from development and wellbeing indicators, which rely on national data accounts (Campos-Matos et al., 2019). Although UN agencies have focused on assessing vulnerability in refugee populations in Lebanon, few of these have systematically included the same data and indicators across the sub-populations residing in Lebanon (Lebanese, Syrian refugees, and Palestinian refugees). The most recent Multiple Indicator Cluster Survey (MICS) for Lebanon dates back to 2009, and the demographic changes that have occurred in the country since are likely to have led to substantial changes in the situation of women and children. In 2015–2016, the UNICEF Lebanon country office designed and implemented a household survey covering all sub-populations resident in Lebanon to generate data on issues relevant to women and children to guide its programs. Considering the lack of other more recent data on the situation of women and children in the country, these data can serve as a baseline of the situation of these populations shortly before the most recent economic and COVID-19 crises, and they can help to identify subgroups of heightened vulnerability to deprivation and its socio-demographic and geographical correlates.

The present study therefore uses the UNICEF Household Survey 2016–2017 to investigate, for the first time, the overlap (co-occurrence) in various forms of deprivation faced by refugee children, as compared to those faced by vulnerable Lebanese children living alongside them in Lebanon, and examines the socio-demographic and geographical correlates of child deprivation in Lebanon. The results presented hereafter serve as baseline data prior to the ongoing economic crisis.

## Methods

### Nationally Representative Data of Four Populations in Lebanon

This analysis used UNICEF's Household Survey data, collected between December 2015 and January 2016. The survey employed specific sampling approaches to obtain nationally representative samples of four respective populations living in Lebanon: Lebanese, Palestinian refugees living in Lebanon, Palestinian refugees from Syria, and Syrian refugees.

In the absence of an updated Lebanese sampling frame for residents of Lebanon, a two-stage stratified cluster sample approach (geographical sampling) was used to collect data on various indicators for Lebanese women and children and included a total sample of 10,053 Lebanese households with data on 38,390 individuals. For the Palestinian refugees living in Lebanon, a sample was extracted from the Palestinian Central bureau of statistics’ (PCBS) most recent census database using a one-stage cluster sampling approach and included a total sample of 4,707 households with 20,282 individuals (Lebanese Palestinian Dialogue Committee et al., 2017). As for Palestinian refugees from Syria, UNICEF referred to the United Nations Relief and Works Agency for Palestine Refugees (UNRWA) list of Palestinian from Syria households and sampled a total of 1,442 households including 8,133 individuals. Lastly, a two-stage stratified sampling was used for Syrian refugees using the United Nations High Commissioner for Refugees (UNHCR) registration database with a total sample of 2,523 households and included 12,661 individuals.

### Dimensions

For this analysis we relied on UNICEF MODA guidelines, (De Neuborg et al., 2012) the available data, and the specificities of the Lebanese context and its various refugee sub-populations to generate the indicators and dimensions. MODA analysis is based on the Convention on the Rights of Child (UNICEF, 1989), the World Summit on Social Development (Midgley, 1995), the Millennium Development Goals (UN General Assembly, 2000) and the Sustainable Development Goals (UN General Assembly, 2015) which inform the construction of the dimensions.

We considered variables related to child survival (health, water, sanitation and overcrowding), child development (education) and child protection (labor, exposure to violence and early marriage) in this analysis based on the Convention on the Rights of Child framework. Two age groups were analyzed separately representing early childhood (children aged 2-4 years) and school age and adolescent children (children aged 6 to 17 years). Children aged 5 were excluded from this analysis as no child-level indicators were available for this age group. Table 1 lists the indicators and dimensions included in the analysis.

**Table 1:** Selected indicators for each dimension, description of deprivation and relevant age group

Dimension	Indicators	Deprivation threshold (Deprived if)	Dimension Age groups (years)	
			2 to 4	6 to 17
Health	Health card	Child aged 24–59 months does not have a health card	X	
	Diphtheria-pertussis-tetanus (DPT) immunization	Child aged 24–59 months has not received all 3 DPT vaccinations		
Education	Compulsory school attendance	Child of compulsory school age (6–14 years old) but not attending school		X
	Primary school attainment	Child beyond primary school age (12–17 years old) with no or incomplete primary education		
Water	Access to improved water source	Child is water deprived if he/she is living in a household's where main source of drinking water is unimproved (unprotected well; unprotected spring; river/dam/lake/pond/stream/canal; tanker truck; cart with small tank; surface water). If the household's main source of drinking water is bottled water, the water source is considered unimproved if the source of main non-drinking water is unimproved	X	X
Sanitation	Access to improved sanitation	Child is deprived if he/she is living in a household with unimproved toilet facility (flush to somewhere else; pit latrine without slab/open pit; bucket toilet; hanging toilet/latrine; no facility/bush/field; other)	X	X
	Shared sanitation	Child is deprived if he/she is living in a household where toilet facility is shared with other households		
Housing	Overcrowding	Child is deprived if he/she is living in a household that includes on average more than three people per sleeping room	X	X
Protection	Domestic violence	Child is deprived if he/she is living in a household d where one child (randomly selected) between 1 and 14 years old experienced any type of physical violence by parents	X	X
	Child labor	Child is deprived if he/she is living in a household where one child (randomly selected) between the ages of 5 and 17 worked for more than 1 h of economic labor		
	Child marriage	Child is deprived if he/she is living in a household where an adolescent female child aged 10–17 is married or in a marital union		

We defined deprivation dimensions at both the child level (health, education) and the household level (water, sanitation, protection). Each dimension includes one or more indicators that have been selected, according to MODA international guidelines, to depict the unique deprivation faced by each age group and sub-population (Chzhen et al., 2014; De Neubourg et al., 2013) (Table 1). Each indicator is measured as a binary variable which takes on the value 1 if a deprivation is presented 0 otherwise. Deprivation thresholds for each indicator were set based on the union approach: a child was found to be deprived in a certain dimension if s/he was deprived in at least one of the indicators of that dimension. The selection of the indicators and thresholds were based on internationally recognized indicators used by MODA and provided by international institutions (WHO, UNESCO, UNICEF, ILO, UN-Habitat).

Deprivation in health was defined as not having a health card or not receiving all 3 DPT immunizations. Education deprivation included the lack of compulsory school attendance or primary school attainment. Water deprivation was defined as lack of access to improved water sources while sanitation deprivation included lack of access to improved sanitation or a household sharing toilet facilities. Housing deprivation was defined as overcrowding (greater than three people sharing a sleeping room). As child labor and exposure to violence data were only collected for one randomly selected child per household, the dimension was converted into a household level variable assuming that any child living in a household where another child is exposed to child labor or violence would be at high risk of child protection concerns. Although we assume that an index child’s siblings may undergo the same treatment or will at least be affected by the violence their sibling is subjected to, this indicator may be an underestimate of exposure if the index child is not reported to be exposed to violence and non-index children (on whom data are not available) are.

All dimensions were given an equal implicit weight as the international children's rights framework does not prioritize particular child rights over others. A total score of deprivation was calculated, following standard MODA methodology (De Neubourg et al., 2013). Following sensitivity analysis, a cutoff point of two deprivations or more was used to define multiple deprivation. Based on MODA recommendations, the cut-off of two or more deprivations is used as the ‘poverty line’ to define deprived children (Hjelm et al., 2016).

### Statistical Analysis

Prevalence of a single deprivation and co-occurrence of multiple deprivations were computed for children aged 2-4 years old and 6-17 years old separately. Adjusted deprivation headcount was also generated by multiplying the headcount ratio (number of children in a specific age group who had two or more deprivation as a percentage of all children in the age group) with average deprivation intensity among the deprived. Socio-demographic and geographical correlates of child multiple deprivations were explored using multivariate logistic regression. The models were adjusted for gender of the child and gender of the head of the household taking into consideration clustering at the household level and sampling weights. Analyses were conducted using the ipfraking procedure for post-stratification (an iterative proportional fitting, adjusting the sampling weights to conform to the specified control totals) in Stata 15 (StataCorp LLC, College Station, TX, US) and online ArcGIS online (Environmental Systems Research Institute).

### Ethical Approval

Ethical approval was granted from the UNICEF Ethics Review Board before the study began. A de-identified dataset was shared with the American University of Beirut and data sharing conditions were governed by a data-transfer agreement.

## Results

### Prevalence of Deprivations by Dimension

This national study included a total of 10,555 Lebanese; 7,106 Palestinian refugees living in Lebanon; 2,768 Palestinian refugees from Syria and 5,891 Syrian children aged 2 to 17 years. In terms of health deprivation, Syrian children aged 2-4 years, experienced the highest prevalence of deprivation (45.8%) as compared to their Lebanese (13.0%), Palestinian refugees living in Lebanon (13.0%), and Palestinian refugees from Syria (11.7%) counterparts (Table 2). As for older children aged 6-17 years, Syrian children also experienced the highest levels of deprivation in education (50.1%) followed by Palestinian refugees from Syria (18.6%), Palestinian refugees living in Lebanon (12.9%) and Lebanese (7.8%) (Table 2).

**Table 2:** Prevalence of deprivation by nationality and age of the child

Deprivations in	2-4 years old					6-17 years old				
	Lebanese	Palestinian refugees living in Lebanon	Palestinian refugees from Syria	Syrian refugees	Total	Lebanese	Palestinian refugees living in Lebanon	Palestinian refugees from Syria	Syrian refugees	Total
*n*	2,563	1,195	568	1,366	5,692	7,312	5,413	1,982	4,000	18,707
Health, %	13.0	13.0	11.7	45.8	24.9	–	–	–	–	–
Education, %	–	–	–	–	–	7.7	13.0	18.6	50.1	23.4
Water, %	1.8	1.2	2.6	26.9	11.0	3.0	1.0	2.6	25.2	8.6
Sanitation, %	2.0	6.9	25.7	18.7	13.5	3.0	5.8	20.0	16.2	10.2
Housing, %	9.8	26.4	47.0	59.6	39.6	18.3	28.4	54.0	70.8	48.2
Protection, %	37.4	66.4	56.6	54.1	56.2	32.0	48.2	48.5	45.9	44.5

Syrian children experienced the highest prevalence of water deprivation compared to Palestinian refugees from Syria, Palestinian refugees living in Lebanon and Lebanese, where Syrian children aged 2-4 years and 6-17 years respectively experienced around 26.9% and 25.2% deprivation, while in other sub-populations water deprivation was below 3%. Deprivation in sanitation in children aged 2-4 years and 6-17 years showed Palestinian children from Syria experienced the highest level of deprivation, followed by Syrian, Palestinian refugees living in Lebanon, and Lebanese children. Similarly, the highest levels of housing deprivation corresponded to children of Syrian nationality followed by Palestinian refugees from Syria, Palestinian refugees living in Lebanon and Lebanese.

As for protection deprivation (combining all 3 measures), it appears that Palestinian children living in Lebanon aged 2-4 years and Palestinian from Syria children aged 6-17 years experienced the highest level of deprivation of 66.4% and 48.5% respectively. These levels of deprivation in protection were found to be largely due to reported exposure of children to domestic violence as opposed to child labor and early marriage.

### Co-occurrence in Deprivations

In terms of co-occurrence of deprivations, Syrian children have the highest prevalence of two or more deprivations in all age groups (68.5% for children aged 2-4y and 65.7% for children aged 6-17y), followed by Palestinian from Syria (46.2% for children aged 2-4y and 45.5% for children aged 6-17), Palestinian refugees living in Lebanon (28.9% for children aged 2-4y and 23.7% for children aged 6-17), with Lebanese children having the lowest prevalence of co-occurrence of deprivations (13.2% for children aged 2-4y and 15.3% for children aged 6-17y).

The adjusted deprivation headcount follows a similar pattern with co-occurrence of two or more deprivation (Syrian 0.36 for children aged 2-4y and 0.46 for children aged 6-17y, Palestinian from Syria 0.21 for children aged 2–4 y and 0.26 for children aged 6-17y, Palestinian refugees living in Lebanon 0.12 for children aged 2–4 y and 0.13 for children aged 6-17y, Lebanese 0.06 for children aged 2–4 y and 0.08 for children aged 6-17y).

After adjusting for gender of the child, gender of the head of the household and governorate, refugee children had higher odds of having two or more deprivations [Palestinian (OR:2.5; 95%CI: 2.0,3.2), Palestinian from Syria (OR:5.2; 95%CI:3.9,6.8), Syrian (OR:10.6; 95%CI: 8.2,13.7)] compared to Lebanese children aged 2-4 years. In a similar pattern, when compared to Lebanese children aged 6-17 years, refugee children had higher odds of having two or more deprivations [Palestinian refugees living in Lebanon (OR:2.0; 95%CI: 1.6;2.4), Palestinian from Syria (OR:5.3; 95%CI:4.2,6.7), Syrian (OR:6.4; 95%CI: 5.2,7.9)] (full models in Appendix Table S1).

**Fig. 1:**
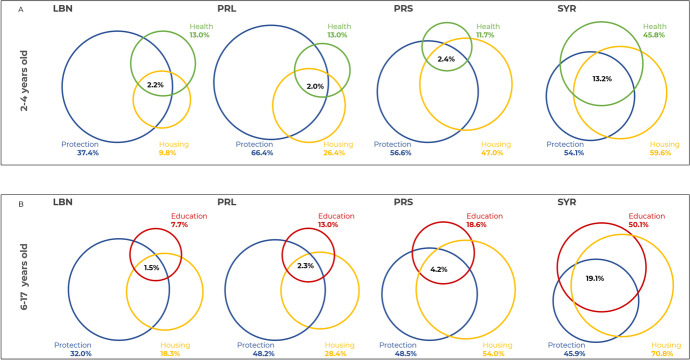
Overlaps in deprivations, LBN (Lebanese), PRL (Palestinian refugees living in Lebanon), PRS (Palestinian refugees from Syria), SYR (Syrian refugees)

Overlapping (co-occurrence) of deprivations were highest across the protection and housing dimensions in children aged 2-4 years and 6-17 years across all four groups. In terms of co-occurrence of three deprivations, Syrian children, aged 2-4 years, had the highest overlap (13.2%) in health, protection, and housing indicators. Similarly, Syrian children aged 6-17 years were shown to have the highest overlap (19.1%) in education, protection and housing indicators as compared to children from other sub-populations (Fig. 1).

### Child Deprivation Inequities as they Relate to Geographic Residency

**Fig. 2:**
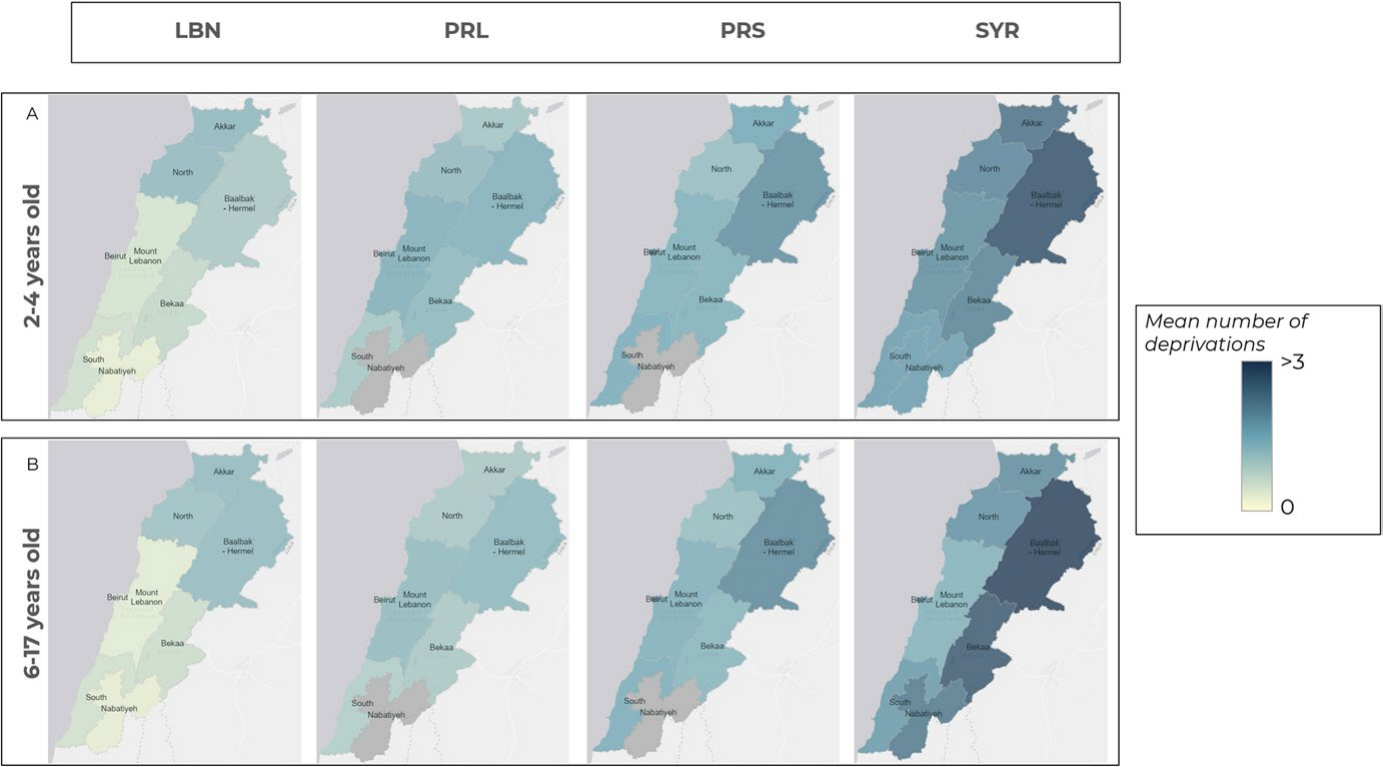
Mean number of deprivations by district, LBN (Lebanese), PRL (Palestinian refugees living in Lebanon), PRS (Palestinian refugees from Syria), SYR (Syrian refugees)

Results indicate considerable differences in the distribution of deprivation across geographical locations. In the North and Akkar governorates, the mean number of deprivations among Lebanese and Palestinian refugees living in Lebanon children were similar (Fig. 2). Compared with 2–4-year-old children living in the capital Beirut, children in the North had 2.49 times the odds of multiple deprivation (95% CI: 1.13, 5.46; *P* = 0.022) (data shown in Appendix S2). Similarly, for older Lebanese children, as compared with children living in Beirut, odds of deprivation were 3.5 in the North (95% CI: 1.9,6.4; *P* < 0.001), 3.05 in Akkar (95% CI: 1.6,5.8 *P* = 0.001) and 3.8 in Baalbak- Hermel (95% CI 2.1- 7.1 *P* < 0.001). The mean number of deprivations among Syrian children is the most widespread with the highest concentration of deprivation existing among those living in the Baalbak -Hermel and Bekaa. Palestinian from Syria children living in Beirut and Baalbak—Hermel have a similar mean number of deprivations to Syrian children. Distribution of deprivation by governorate is almost unchanged between the two age groups for the same subpopulation.

### Child Deprivation Inequities as they Relate to Maternal Education

**Fig. 3:**
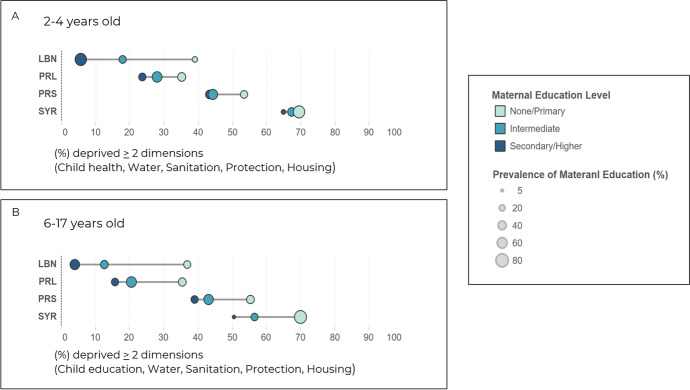
Inequities as they relate to maternal education, LBN (Lebanese), PRL (Palestinian refugees living in Lebanon), PRS (Palestinian refugees from Syria), SYR (Syrian refugees)

The prevalence of multiple deprivation by maternal education and the distance between maternal education levels were plotted using equity plots for the different sub-populations of children (Fig. 3). This analysis allowed us to compare child inequalities as they relate to maternal education. For Lebanese and Palestinian children living in Lebanon aged 2-4 years, multiple deprivation varied by maternal education. The analysis revealed that children from Lebanese nationalities aged 2-4 years were most deprived (40%) if their mothers had attained none/primary and least deprived (5%) if their mothers had secondary/higher education (Fig. 3A). In fact, higher maternal education (intermediate and secondary plus) was significantly associated with lower odds of having at least two concurrent deprivations among Lebanese and Palestinian children living in Lebanon aged 2-4y (Table 3). Nonetheless, child deprivation among Syrian and Palestinian from Syria children in this age group remained high and unchanged despite different maternal education levels (Fig. 3A).

**Table 3:** Association between child experiencing multiple deprivation and maternal education, by nationality

		Lebanese			Palestinian refugees living in Lebanon			Palestinian refugees from Syria			Syrian refugees		
		OR	95% CI	*P*	OR	95% CI	*P*	OR	95% CI	*P*	OR	95% CI	*P*
Age 2-4 years	Maternal education												
	None/primary	–			–			–			–		
	Intermediate	0.5	[0.3–0.7]	< 0.001	0.8	[0.6–1.1]	0.205	0.6	[0.4–1.0]	0.061	0.9	[0.6–1.3]	0.461
	Secondary plus	0.2	[0.1–0.3]	< 0.001	0.6	[0.4–0.9]	0.010	0.6	[0.4–1.1]	0.089	0.8	[0.4–1.4]	0.390
Age 6-17 years	Maternal education												
	None/primary	–			–			–			–		
	Intermediate	0.3	[0.2–0.5]	< 0.001	0.5	[0.4–0.6]	< 0.001	0.6	[0.4–0.8]	0.001	0.5	[0.4–0.7]	< 0.001
	Secondary plus	0.1	[0.1–0.2]	< 0.001	0.3	[0.3–0.5]	< 0.001	0.5	[0.3–0.7]	< 0.001	0.4	[0.2–0.7]	< 0.001

Models adjusted for gender of the child, gender of the head of the household and governorate, clustered at the household level (full model in Appendix S2)

A similar pattern of deprivation was found among children aged 6-17 years; whereby maternal educational level was found to be associated with child deprivation levels across all sub-populations (Fig. 3B). For instance, among Syrian refugee children, a 20% difference in deprivation can be found as a function of lower and higher maternal educational levels (Fig. 3B). In this age group, higher maternal education (completed intermediate education) was consistently and significantly associated with lower odds of having at least two concurrent deprivations across all sub-populations (Table 3).

## Discussion

This analysis is the first to compare child wellbeing indicators across Lebanese nationals and three different refugee sub-populations in Lebanon prior to the most recent economic crisis. Our study shows large inequities by sub-population and geographic location and highlights the strong association found between maternal education and multiple deprivation in children living in Lebanon, particularly for children of school age. For younger children, the association is slightly weaker for the more recent refugees from Syria (Palestinian refugees from Syria and Syrian).

Results highlight overlaps between poor housing (overcrowding) and exposure to violence against children in Syrian and Palestinian refugee children, in turn indicating the need to simultaneously focus on housing improvements and protection programs. For this, avoiding programs that work in silos and instead promoting a multisectoral approach which recognizes the need to engage various governmental sectors (such as education, law enforcement, health and others) becomes warranted to concurrently tackle multiple deprivations and in turn ensure proper child development (Strong et al., 2021). In Brazil for instance, it is well documented that the inter-sectoral coordination approach of improving maternal education, income of families, access and availability of health care services and improvement of infrastructure, specifically sanitation, led to a drop in stunting from 13.5% to 6.8% between 1996 and 2006/2007 (del Carmen Casanovas et al., 2013; Monteiro et al., 2009). Similarly, a community development intervention in Nepal showed that children in the multisectoral intervention groups (which included community development initiatives, livestock training, and nutrition education) demonstrated better feeding practices at end-line, as well as improvements in household wealth and hygiene practices (Miller et al., 2020).

Geographical disparities point to areas where children are particularly vulnerable in Lebanon. The geographical areas with highest deprivation are also the regions where the uprisings of October 2019 were most intense (Akkar-North and Baalbak-Hermel) (Bérangère Pineau Soukkarieh et al., 2020). In October 2019, civil movements took place across Lebanon for several months to protest the economic and political situation in Lebanon (Bérangère Pineau Soukkarieh et al., 2020). These areas have substantial deficits in fundamental socio-economic infrastructures, which consequently translate to gaps in living standards and ultimately higher levels of child poverty and deprivation(Ogwumike & Ozughalu, 2018). A comprehensive and sustainable plan for adequate investment in the development of these two regions in terms of social welfare, education, sanitation, water supply and more is key to improving child wellbeing outcomes (Padda & Hameed, 2018).

Maternal education has been widely recognized as one of the most pivotal mediators in child health and educational outcomes (Vikram et al., 2012). In our study, we report significant associations between maternal education and children's experience with multiple deprivations. It is well established that children raised to mothers with higher educational attainment have lower levels of both health and education deprivations (Güneş, 2015). Despite all efforts, more than half of Syrian children, mainly adolescents and youth, are still out of school. Since 2014, UNHCR, the Ministry of Education and Higher Education and UNICEF have developed and implemented an education response plan, Reaching All Children with Education to ensure the access of disadvantaged Syrian children to learning opportunities (MEHE, 2014). The program invested enormous resources into accommodating a large number of children within its system through creating two separate yet complementary shifts in public schools. Further investment in adolescent girls’ education has an intergenerational effect, and will be particularly important to ensure that adolescent Syrian refugee girls reach and complete secondary education to break the cycle of child deprivation (Patton et al., 2016).

The study also indicates that large inequities across sub-populations resulting from their unequal legal standing and the difference in the infrastructures that provide humanitarian and development services to the different sub-populations. Our study indicates that among the most recently displaced populations due to the Syrian conflict (Palestinian refugees from Syria and Syrian). Palestinian from Syria children fare better than their Syrian counterparts on various indicators. Deprivations in health and education are comparatively low in Palestinian from Syria children as they have been incorporated into UNRWA’s well established health and education systems largely protecting them from poor health and education outcomes (Jamal et al., 2020). UNRWA offers free comprehensive primary health care to infants and children, including growth monitoring, immunization, screening for disabilities, oral health checkups, among others. Additionally, it provides free education to 31,706 Palestinian and 5,254 Palestinian from Syria in its 65 schools in Lebanon (UNRWA). High deprivation in health and education indicators for Syrian refugees indicate that children face structural barriers to wellbeing.

As economic pressures drive more Lebanese to the public sector for health and education, there is a risk of these being overwhelmed and failing vulnerable populations, both Lebanese and Syrian. Further investments in strengthening public health and public education are essential given the current economic crisis and the risk of huge potential migration into the public system.

This study highlights the stark inequities that exist across child subpopulations living side-by-side in Lebanon and illustrates the importance of considering displacement as a social determinant of child health and the importance of including refugee populations in development reporting frameworks. The results indicate the importance of not pooling data from different subpopulations in analyses and disaggregating subpopulation data, to avoid masking deprivations faced by specific vulnerable subpopulations that have different legal rights in the country. This is the first study to collect representative data from these four sub-populations in parallel, allowing granular analyses to inform programs and policies in Lebanon. This study forms an important baseline that can be used to estimate and therefore mitigate the impacts of the current economic crisis on child wellbeing.

The study is limited by the data collected in the survey on which it relies, which excludes indicators of other child wellbeing dimensions such as nutrition, leisure, freedom of expression amongst others. The survey also does not include health level data for children 6-17 years old and completely lacks child level indicators for children aged 5 years. The lack of child-specific indicators for children older than 5 years old has been noted in the literature and child-reported indicators that reflect children’s own lived experience and voice should be considered in future surveys (Strong et al., 2021). Our analysis focuses on overlaps in two or more deprivations, but variability exists in this category and such an aggregation may mask extremes of deprivation. While the use of a union approach has its limitations, it is motivated by the rights-based approach used in MODA. Each indicator is considered equally important for a child within any dimension. The MODA methodology used in the analysis often relies on household-level data to indicate households where children experience deprivation and thus the MODA approach is child responsive, it is not child centered. The limited availability of child centered indicators highlights the need to include indicators that report on children’s own experiences of deprivation; one such example is the recently developed child food insecurity scale(Frongillo et al., 2022; Jamaluddine et al., 2019). Children in the household exposure to violence may be underestimated if the index child is not reported to be exposed to violence and non-index children are. It is worth noting that deprivation scores of younger children and older children in the same subpopulation are not comparable as the results rely on different indicators for younger and older children. The present analysis provides a snapshot of the situation of children in Lebanon prior to the onset of the most recent economic crisis, which began in 2019. However, given the dynamic nature of economic conditions, a follow-up assessment is necessary to monitor changes in the deprivations experienced by children and to inform the targeting of social protection programs aimed at mitigating their vulnerability.

## Conclusion

Refugee children face structural barriers to wellbeing that include access to adequate housing, protection, health, and education, and are more likely to be deprived of child rights than other vulnerable children living in Lebanon. Addressing these structural barriers will be essential to prevent human capital losses in a whole generation of vulnerable children living in Lebanon. The findings of this study can contribute to guiding interventions in Lebanon and the development of a long-term strategic action plan for the improvement of national and refugee child wellbeing in the country given the current economic crisis.

## Supplementary Information

Below is the link to the electronic supplementary material.

Supplementary file1 (DOCX 32 KB)

## Data Availability

UNICEF is the main custodian of the data. Data can be made are available from UNICEF Lebanon for researchers who meet the criteria for access to confidential data.
