# Supplementary material for: Inequalities in Wellbeing in Lebanese Children and Different Refugee Subpopulations: A Multidimensional Child Deprivation Analysis
Source: Child Indic Res. 2023 Jun 6:1–19. Online ahead of print. doi: 10.1007/s12187-023-10040-2 (PMC10241607; doi:10.1007/s12187-023-10040-2)
Supplement: Supplementary file 1 — Supplementary file1 (DOCX 32 KB) [file 12187_2023_10040_MOESM1_ESM.docx]

**Table S1. Association between child experiencing multiple deprivations and nationality**

|  | Age 2-4 years old | | | Age 6-17 years old | | |
| --- | --- | --- | --- | --- | --- | --- |
|  | OR | 95% CI | P | OR | 95% CI | P |
| **Cohort** |  |  |  |  |  |  |
| Lebanese residents | - |  |  | - |  |  |
| Syrian Registered Refugees | 10.6 | [8.2-13.7] | <0.001 | 6.4 | [5.2-7.9] | <0.001 |
| Palestine Refugees in Lebanon (PRL) | 2.5 | [2.0-3.2] | <0.001 | 2.0 | [1.6-2.4] | <0.001 |
| Palestine Refugees from Syria (PRS) | 5.2 | [3.9-6.8] | <0.001 | 5.3 | [4.2-6.7] | <0.001 |
| **Sex** |  |  |  |  |  |  |
| Male | - |  |  |  |  |  |
| Female | 1.1 | [0.9-1.2] | 0.538 | 0.9 | [0.9-1.02] | 0.139 |
| **Maternal education** |  |  |  |  |  |  |
| None/primary | - |  |  | - |  |  |
| Intermediate | 0.8 | [0.6-1.0] | 0.016 | 0.5 | [0.4-0.5] | <0.001 |
| Secondary plus | 0.6 | [0.4-0.7] | <0.001 | 0.3 | [0.2-0.4] | <0.001 |
| **Sex of household head** |  |  |  |  |  |  |
| Male | - |  |  | - |  |  |
| Female | 1.5 | [1.1-2.1] | 0.013 | 1.1 | [0.9-1.3] | 0.464 |
| **Region** |  |  |  |  |  |  |
| Beirut | - |  |  | - |  |  |
| Mount Lebanon | 0.8 | [0.5-1.4] | 0.494 | 0.7 | [0.5-1.0] | 0.029 |
| North | 0.8 | [0.5-1.3] | 0.410 | 1.1 | [0.8-1.5] | 0.613 |
| Akkar | 0.7 | [0.4-1.1] | 0.152 | 1.0 | [0.7-1.4] | 0.915 |
| Bekaa | 0.7 | [0.4-1.2] | 0.250 | 1.9 | [1.3-2.6] | <0.001 |
| South | 0.5 | [0.3-0.9] | 0.015 | 0.8 | [0.6-1.1] | 0.130 |
| El Nabatieh | 0.4 | [0.2-0.6] | <0.001 | 1.1 | [0.7-1.5] | 0.772 |
| Baalbek El Hermel | 1.1 | [0.7-1.9] | 0.602 | 2.4 | [1.7-3.4] | <0.001 |

| Table S2. **Association between child experiencing multiple deprivations and maternal education, child sex, sex of the head of the household and region, by nationality.** |  | LBN | | | PRL | | | PRS | | | SYR | | |
| --- | --- | --- | --- | --- | --- | --- | --- | --- | --- | --- | --- | --- | --- |
|  |  | OR | 95% CI | P | OR | 95% CI | P | OR | 95% CI | P | OR | 95% CI | P |
| Age 2-4 years old | **Maternal education** |  |  |  |  |  |  |  |  |  |  |  |  |
|  | None/primary | - |  |  | - |  |  | - |  |  | - |  |  |
|  | Intermediate | 0.5 | [0.3-0.7] | <0.001 | 0.8 | [0.6-1.1] | 0.205 | 0.6 | [0.4-1.0] | 0.061 | 0.9 | [0.6-1.3] | 0.461 |
|  | Secondary plus | 0.2 | [0.1-0.3] | <0.001 | 0.6 | [0.4-0.9] | 0.010 | 0.6 | [0.4-1.1] | 0.089 | 0.8 | [0.4-1.4] | 0.390 |
|  | **Sex** |  |  |  |  |  |  |  |  |  |  |  |  |
|  | Male | - |  |  | - |  |  | - |  |  | - |  |  |
|  | Female | 0.9 | [0.7-1.1] | 0.316 | 1.2 | [0.9-1.5] | 0.252 | 0.8 | [0.6-1.1] | 0.176 | 1.2 | [0.9-1.6] | 0.249 |
|  | **Sex of household head** |  |  |  |  |  |  |  |  |  |  |  |  |
|  | Male | - |  |  | - |  |  | - |  |  | - |  |  |
|  | Female | 1.2 | [0.6-2.7] | 0.618 | 2.1 | [1.1-4.2] | 0.032 | 1.7 | [1.0-2.9] | 0.048 | 1.0 | [0.7-1.7] | 0.860 |
|  | **Region** |  |  |  |  |  |  |  |  |  |  |  |  |
|  | Beirut | - |  |  | - |  |  | - |  |  | - |  |  |
|  | Mount Lebanon | 0.7 | [0.3-2.2] | 0.622 | 1.7 | [0.5-5.9] | 0.435 | 0.2 | [0.4-0.7] | 0.015 | 1.0 | [0.5-1.9] | 0.992 |
|  | North | 2.5 | [1.1-5.5] | 0.022 | 1.1 | [0.3-3.9] | 0.913 | 0.1 | [0.0-0.4] | 0.002 | 1.2 | [0.7-2.0] | 0.562 |
|  | Akkar | 2.2 | [1.0-4.8] | 0.060 | 0.5 | [0.1-1.9] | 0.317 | 0.2 | [0.0-0.9] | 0.037 | 1.4 | [0.8-2.5] | 0.188 |
|  | Bekaa | 0.9 | [0.4-2.0] | 0.802 | 1.7 | [0.4-7.4] | 0.450 | 0.1 | [0.0-0.6] | 0.010 | 1.0 | [0.6-1.6] | 0.974 |
|  | South | 0.6 | [0.2-1.5] | 0.241 | 0.7 | [0.2-2.4] | 0.543 | 0.2 | [0.0-0.7] | 0.016 | 0.6 | [0.4-1.2] | 0.144 |
|  | El Nabatieh | 0.4 | [0.2-1.0] | 0.057 | - |  |  | - |  |  | 0.6 | [0.4-0.9] | 0.023 |
|  | Baalbek El Hermel | 1.4 | [0.7-3.1] | 0.360 | 2.7 | [0.6-12.6] | 0.200 | 0.2 | [0.1-1.2] | 0.089 | 1.5 | [0.9-2.5] | 0.095 |
| Age 6-17 years old | **Maternal education** |  |  |  |  |  |  |  |  |  |  |  |  |
|  | None/primary | - |  |  | - |  |  | - |  |  | - |  |  |
|  | Intermediate | 0.3 | [0.2-0.5] | <0.001 | 0.5 | [0.4-0.6] | <0.001 | 0.6 | [0.4-0.8] | 0.001 | 0.5 | [0.4-0.7] | <0.001 |
|  | Secondary plus | 0.1 | [0.1-0.2] | <0.001 | 0.3 | [0.3-0.5] | <0.001 | 0.5 | [0.3-0.7] | <0.001 | 0.4 | [0.2-0.7] | <0.001 |
|  | **Sex** |  |  |  |  |  |  |  |  |  |  |  |  |
|  | Male | - |  |  | - |  |  | - |  |  | - |  |  |
|  | Female | 0.8 | [0.6-1.0] | 0.022 | 0.9 | [0.8-1.1] | 0.354 | 0.9 | [0.7-1.0] | 0.086 | 1.1 | [0.9-1.3] | 0.344 |
|  | **Sex of household head** |  |  |  |  |  |  |  |  |  |  |  |  |
|  | Male | - |  |  | - |  |  | - |  |  | - |  |  |
|  | Female | 1.0 | [0.5-2.0] | 0.929 | 1.0 | [0.7-1.4] | 0.951 | 1.1 | [0.8-1.6] | 0.550 | 1.0 | [0.7-1.4] | 0.979 |
|  | **Region** |  |  |  |  |  |  |  |  |  |  |  |  |
|  | Beirut | - |  |  | - |  |  | - |  |  | - |  |  |
|  | Mount Lebanon | 0.3 | [0.1-0.8] | 0.021 | 1.4 | [0.7-3.1] | 0.343 | 0.4 | [0.2-0.9] | 0.023 | 0.4 | [0.3-0.6] | <0.001 |
|  | North | 3.5 | [1.9-6.4] | <0.001 | 1.0 | [0.5-2.2] | 0.985 | 0.3 | [0.1-0.7] | 0.005 | 0.9 | [0.6-1.4] | 0.566 |
|  | Akkar | 3.1 | [1.6-5.8] | 0.001 | 0.9 | [0.4-2.0] | 0.739 | 0.4 | [0.2-1.2] | 0.097 | 0.8 | [0.5-1.2] | 0.314 |
|  | Bekaa | 1.3 | [0.7-2.5] | 0.480 | 1.0 | [0.4-2.5] | 0.954 | 0.3 | [0.1-0.8] | 0.020 | 2.6 | [1.7-4.0] | <0.001 |
|  | South | 2.0 | [0.9-4.2] | 0.079 | 0.8 | [0.4-1.6] | 0.461 | 0.5 | [0.2-1.1] | 0.068 | 0.7 | [0.4-1.1] | 0.145 |
|  | El Nabatieh | 0.7 | [0.3-1.5] | 0.306 | - |  |  | - |  |  | 1.6 | [1.0-2.5] | 0.029 |
|  | Baalbek El Hermel | 3.8 | [2.1-7.1] | <0.001 | 1.7 | [0.7-4.3] | 0.265 | 1.0 | [0.4-2.8] | 0.951 | 2.4 | [1.6-3.7] | <0.001 |
